# Regulatory logic of the *Chlamydomonas* CO_2_-concentrating mechanism: coupling carbon flux, energy supply, and pyrenoid architecture

**DOI:** 10.3389/fpls.2026.1882169

**Published:** 2026-07-08

**Authors:** Daisuke Shimamura, Takashi Yamano

**Affiliations:** 1 Graduate School of Biostudies, Kyoto University, Kyoto, Japan; 2 RIKEN Center for Sustainable Resource Science, Yokohama, Japan

**Keywords:** carbonic anhydrase, CCM regulation, *Chlamydomonas reinhardtii*, CO_2_ sensing, CO_2_-concentrating mechanism, post-translational regulation, pyrenoid

## Abstract

The algal CO_2_-concentrating mechanism (CCM) elevates CO_2_ around Rubisco, the CO_2_-fixing enzyme, through the coordinated action of inorganic carbon (Ci) uptake systems, carbonic anhydrases (CAs), and CO_2_-retention mechanisms, thereby maintaining photosynthetic efficiency under CO_2_-limiting conditions. Although CCMs occur across diverse aquatic photosynthetic organisms, the green alga *Chlamydomonas reinhardtii* has become the principal model for dissecting the molecular regulation of the eukaryotic algal CCM, and this review focuses on regulatory mechanisms established in this organism. Recent studies have clarified energy-supply networks that support the CCM, including cyclic and pseudo-cyclic electron transfer, chloroplast–mitochondrial cooperation, and mitochondrial relocation toward the cell periphery during CCM induction, as well as multilayered regulatory circuits that tune CCM function across timescales. These circuits include CCM1/CIA5-dependent transcriptional control, Ca^2+^-binding protein CAS-dependent chloroplast Ca^2+^ signaling, protein relocalization, KEY1-dependent post-translational control of pyrenoid architecture, and active repression by the CCM1/CIA5-binding protein CBP1. However, these advances are still often discussed separately, and an explicit systems-level synthesis of carbon flux, energy supply, pyrenoid architecture, and regulation remains limited. Here, we describe the *Chlamydomonas* CCM as a coupled system in which Ci transport routes, CA deployment, CO_2_-retention mechanisms, electron-transfer pathways, and regulatory circuits are dynamically coordinated according to CO_2_ availability. We also discuss how far these regulatory layers may extend beyond *Chlamydomonas* within green algae, emphasizing the limited conservation of CCM1/CIA5- and LCR1-type transcriptional regulators and the presence of CAS-linked chloroplast Ca^2+^ signaling in both *Chlamydomonas* and land plants. Finally, we consider how this regulatory perspective can inform future CCM engineering, arguing that crop-oriented designs may ultimately need not only transferred pyrenoid components but also regulatory strategies that dynamically tune CCM activity according to carbon and energy status.

## Introduction

The CO_2_-concentrating mechanism (CCM) is an active CO_2_-delivery system that elevates CO_2_ around Rubisco, the CO_2_-fixing enzyme, to compensate for its slow carboxylation rate and competition with O_2_. In aquatic photosynthetic organisms, CCMs are implemented by different molecular components, but many rely on a shared functional logic that combines active inorganic carbon (Ci) accumulation, localized conversion of HCO_3_^−^ to CO_2_ by carbonic anhydrases (CAs), and CO_2_ retention or recapture. These principles have been established over several decades through studies of diverse algae and other photosynthetic microorganisms (Giordano et al., 2005; Raven et al., 2014; Reinfelder, 2011).

Among green algae, the molecular regulation of the CCM has been investigated most deeply in *Chlamydomonas reinhardtii*. This review therefore uses the *Chlamydomonas* CCM as its primary framework and focuses on regulatory mechanisms that have been experimentally defined in this organism. Unless explicitly stated otherwise, mechanistic discussions of CCM regulation refer to *Chlamydomonas*. Evidence from other green algae is discussed only where it helps evaluate whether *Chlamydomonas*-derived regulatory principles may extend beyond this model.

The structure, assembly, and engineering potential of the *Chlamydomonas* pyrenoid have been reviewed extensively elsewhere (Barrett et al., 2021; He et al., 2023; Catherall et al., 2025). Therefore, rather than providing another detailed review of pyrenoid architecture, we focus here on the regulatory logic that coordinates carbon flux, energy supply, protein localization, Ca^2+^-linked signaling, post-translational modification, and transcriptional induction or repression in response to CO_2_ availability.

Within this *Chlamydomonas* framework, the CCM is not simply a collection of Ci transport proteins and CAs. Its operation requires photosynthetic ATP under CO_2_-limiting conditions (Giordano et al., 2005; Raven et al., 2014) and meeting this energy cost requires alternative electron-transfer pathways within the chloroplast as well as chloroplast–mitochondrial cooperation. CCM components must also be coordinated across timescales from seconds to hours in response to fluctuating environmental CO_2_. A full understanding of the CCM therefore requires a perspective that treats carbon flux, energy supply, pyrenoid architecture, and regulation as interconnected parts of a coupled system.

Recent advances in *Chlamydomonas* now allow such an integrated view. On the energy side, Burlacot et al. (2022) demonstrated that cyclic and pseudo-cyclic electron transfer are essential for driving the CCM, and Findinier et al. (2024) showed that mitochondria relocate to the cell periphery during CCM induction, consistent with respiratory support for CCM performance. On the regulatory side, CCM1/CIA5 controls the induction of many CCM genes under CO_2_-limiting conditions (Fang et al., 2012; Fukuzawa et al., 2001; Miura et al., 2004; Xiang et al., 2001). This transcriptional framework has been expanded by studies of low-CO_2_ inducible protein B and C (LCIB–LCIC) relocalization (Toyokawa et al., 2020; Yamano et al., 2010, 2022), CAS-dependent Ca^2+^-linked feedback (Shimamura et al., 2023; Wang et al., 2016), *in vitro* biochemical evidence for CCM1/CIA5 DNA binding (Chen and Spalding, 2026), active repression by the nuclear protein CBP1 (Shimamura et al., 2026), condensate-level control by the pyrenoid kinase KEY1 (He et al., 2026), and the joint operation of CCM induction and photorespiration during acclimation to CO_2_-limiting conditions (Dao et al., 2025). Together, these studies show that the *Chlamydomonas* CCM is an environmentally responsive system in which transport, energy supply, pyrenoid architecture, transcriptional regulation, and post-translational control are tightly coupled.

**Figure 1:**
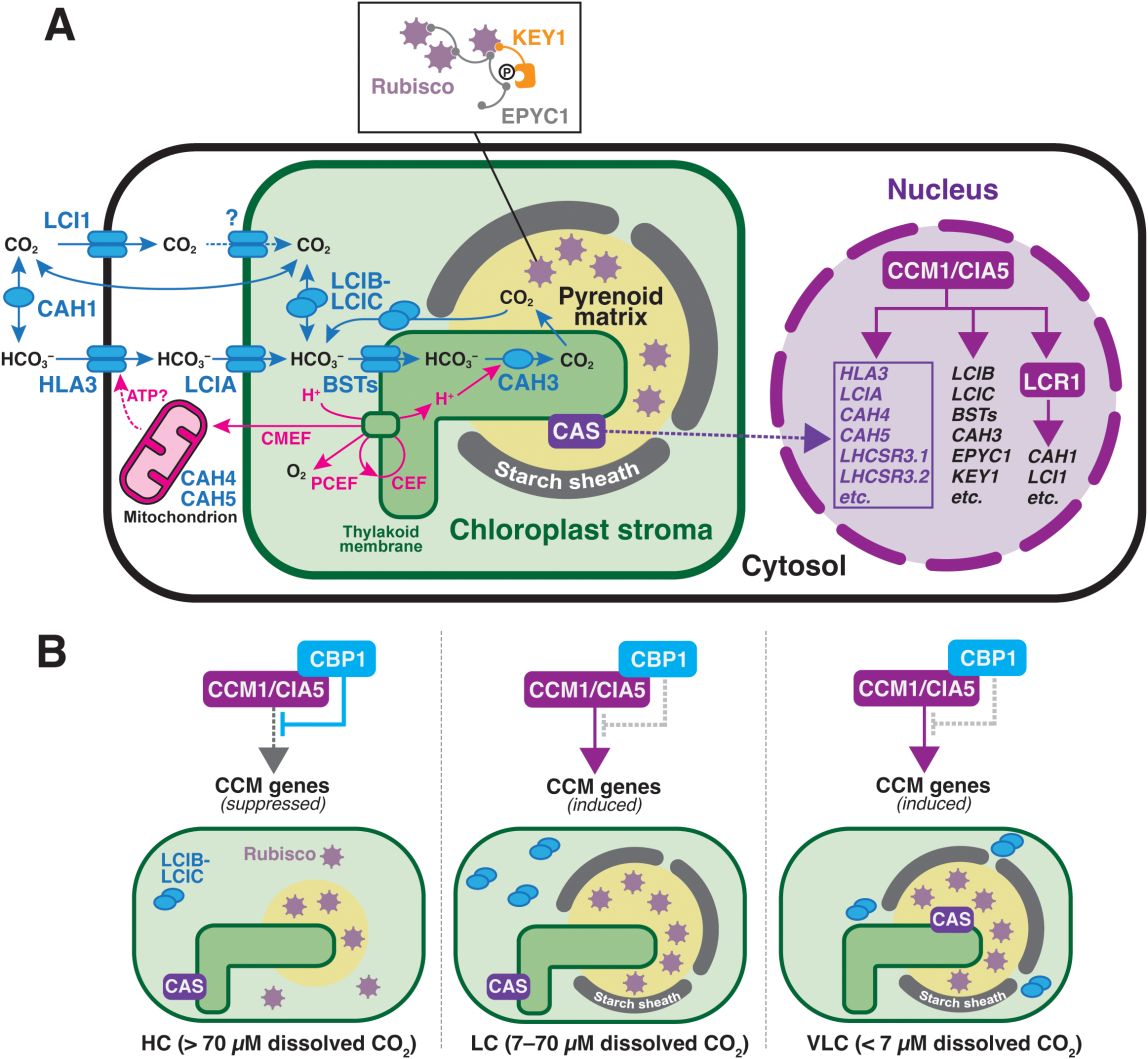
Integrated overview of the *Chlamydomonas* CCM. **(A)** Schematic of a *Chlamydomonas* cell showing the coupling of Ci transport, energy supply, and regulation. Blue arrows indicate Ci/CO_2_ flux, magenta arrows indicate energy-related processes, and purple arrows indicate regulatory pathways. The figure integrates CCM modules that are engaged to different extents depending on CO_2_ availability. Under VLC conditions, HCO_3_^−^ is taken up through HLA3, transported across the chloroplast envelope by LCIA, and delivered to the thylakoid lumen through BST1–3, where CAH3 dehydrates HCO_3_^−^ to CO_2_ near the Rubisco-rich pyrenoid matrix. Under LC conditions, LCI1 and CAH1 support an alternative CO_2_-entry route. LCIB–LCIC localizes around the pyrenoid periphery under VLC conditions and is proposed to recapture CO_2_ leaking from the pyrenoid. Energy-related pathways include cyclic electron flow around photosystem I (CEF), flavodiiron protein-dependent pseudo-cyclic electron flow/O_2_ photoreduction (PCEF), and chloroplast-to-mitochondrion electron flow (CMEF). CEF and PCEF contribute to thylakoid ΔpH formation, supporting ATP synthesis and lumenal CAH3 activity, whereas CMEF and mitochondrial relocation toward the cell periphery may support non-thylakoid Ci uptake steps through respiratory ATP production. KEY1 phosphorylates EPYC1 to regulate pyrenoid condensate dynamics, and CAS mediates chloroplast-to-nucleus signaling that supports CCM gene expression. CCM1/CIA5 controls the induction of many CCM-associated genes under CO_2_-limiting conditions. However, except for the LCR1 branch, which regulates CAH1, LCI1, and related target genes, whether CCM1/CIA5 directly activates the listed genes or acts through additional transcription factors remains unresolved. Solid arrows indicate established pathways, whereas dashed arrows indicate proposed or incompletely resolved steps. **(B)** Schematic summary of how the regulatory state and chloroplast protein localization of the CCM change under HC, LC, and VLC conditions, as operationally defined in this review. Under HC conditions, CO_2_ enters mainly by diffusion, and CBP1 interacts with CCM1/CIA5 to repress a large subset, but not all, of CCM1/CIA5-dependent genes, thereby keeping CCM induction weak. Under LC conditions, CBP1-mediated repression is reduced and CCM1/CIA5-dependent gene induction increases, while LCIB–LCIC remains largely stromal. Under VLC conditions, LCIB–LCIC relocalizes to the pyrenoid periphery, where it is proposed to contribute to CO_2_ leak recapture, and CAS is shown near the pyrenoid to indicate CAS-dependent chloroplast-to-nucleus signaling. Purple arrows indicate CCM1/CIA5-dependent induction rather than proven direct activation of all CCM genes.

In this review, we use the *Chlamydomonas* CCM as the primary framework for integrating driving mechanism, energy supply, and multilayered regulation. We first outline how Ci transport routes, CA deployment, pyrenoid architecture, and CO_2_-retention mechanisms are rewired according to CO_2_ availability. We then discuss how electron-transfer pathways and chloroplast–mitochondrial cooperation meet the energetic demands of Ci transport and localized CO_2_ generation. Finally, we examine how transcriptional control, protein relocalization, Ca^2+^-linked feedback, condensate-level post-translational regulation, and transcriptional repression coordinate these components. We close by considering how the *Chlamydomonas* regulatory architecture may or may not extend to other green algae, and what this means for future engineering applications. **Figure 1** summarizes this framework: **Figure 1A** integrates Ci transport, energy supply, and regulatory modules, whereas **Figure 1B** illustrates how regulatory state and selected chloroplast protein localizations change across high CO_2_ (HC), low CO_2_ (LC), and very-low CO_2_ (VLC) conditions.

## Operational definitions of HC, LC, and VLC in *Chlamydomonas*

Throughout this review, we use the terms HC, LC, and VLC as operational acclimation states rather than as universally fixed gas-phase values. In controlled gas-phase experiments, these states are often separated clearly by the supplied CO_2_ concentration. For example, cells grown on plates or in chambers may be exposed to HC conditions such as 5% CO_2_, LC conditions approximating air-level CO_2_ around 0.04%, or VLC conditions such as 0.01% CO_2_. Such gas-phase definitions are particularly useful for growth assays because the imposed atmospheric CO_2_ concentration is well controlled and reproducible (Toyokawa et al., 2020).

In liquid culture, however, the distinction between LC and VLC is more difficult to define from the supplied gas phase alone. Dissolved CO_2_ depends not only on the aerated CO_2_ concentration but also on pH, total dissolved Ci, gas exchange, buffering capacity, cell density, and CA activity. Therefore, a nominal gas-phase CO_2_ concentration does not always correspond directly to the CO_2_ concentration experienced by the cell. For this reason, when discussing liquid-culture experiments, we distinguish LC and VLC primarily by the calculated or measured dissolved CO_2_ concentration and by physiological markers of the acclimation state.

A useful operational boundary was provided by LCIB localization. LCIB is dispersed in the chloroplast stroma under HC and LC conditions but relocates to the pyrenoid periphery under VLC conditions. This reversible localization switch is controlled by CO_2_ concentration, with approximately 7 µM dissolved CO_2_ as the boundary between LC and VLC. In that framework, VLC corresponds to <7 µM dissolved CO_2_, LC to approximately 7–70 µM dissolved CO_2_, and HC to >70 µM dissolved CO_2_ in liquid-culture assays (Yamano et al., 2022). This threshold also matches physiological behavior of LCIB-related mutants and oxygen-evolution responses, supporting the view that LC and VLC are distinct acclimation states rather than simply different degrees of a single CO_2_-limiting state (Wang and Spalding, 2014; Yamano et al., 2022).

Accordingly, in this review, “CO_2_-limiting conditions” is used as a broad term that includes both LC and VLC. “LC” refers to moderate CO_2_ limitation, including air-level CO_2_ conditions, in which the LCI1–CAH1 route contributes substantially to CO_2_ uptake and LCIB–LCIC remains largely stromal. LCIB-dependent recapture may still contribute to Ci affinity, but pyrenoid-peripheral enrichment of LCIB–LCIC is a hallmark of VLC rather than LC. “VLC” refers to more severe CO_2_ limitation, typically below the ∼7 µM dissolved CO_2_ boundary in liquid culture, in which LCIB–LCIC relocalizes to the pyrenoid periphery and HCO_3_^−^ uptake through the HLA3–LCIA–BST–CAH3 axis becomes especially important. We use “HC” for high-CO_2_-supplied conditions in which the CCM is repressed or only weakly active. When possible, we specify whether a statement refers to gas-phase CO_2_, calculated dissolved CO_2_, or a biological marker such as LCIB localization. These operational features are summarized in **Table 1**.

**Table 1:** Operational features of HC, LC, and VLC acclimation states in *Chlamydomonas reinhardtii*.

Feature	HC	LC	VLC
Dissolved CO_2_ range	>70 µM	7–70 µM	<7 µM
Gas-phase example	5% CO_2_	~0.04% CO_2_	~0.01% CO_2_
Transcriptional state	Weak CCM induction; CBP1 restrains many, but not all, CCM1/CIA5-dependent genes	CCM1/CIA5-dependent induction active; CBP1 repression reduced	Strong CCM1/CIA5-dependent induction
Major Ci route	CO_2_ diffusion predominates	LCI1–CAH1-supported CO_2_ entry	HLA3–LCIA–BSTs–CAH3 HCO_3_^−^ axis
LCIB–LCIC/CAS state	LCIB–LCIC stromal; CAS basal	LCIB–LCIC stromal; CAS signaling active	LCIB–LCIC at pyrenoid periphery; pyrenoid-associated CAS
Energy demand and support	Low CCM-specific demand; basal metabolism	Increased demand; CEF/PCEF support ΔpH and ATP supply	High demand; CEF/PCEF, CMEF, and mitochondria are especially important
ATP-dependent or linked steps	No major active Ci uptake emphasized	LCI1–CAH1 route not known to require direct ATP hydrolysis	HLA3 is clearest ATP-dependent candidate; LCIA/BST likely gradient-dependent; CAH3 depends on lumenal ΔpH

Ranges refer to dissolved CO_2_ in liquid culture. CEF, cyclic electron flow; PCEF, pseudo-cyclic electron flow; CMEF, chloroplast-to-mitochondrion electron flow.

## Driving mechanism of the CCM

In *Chlamydomonas*, efficient CCM operation can be described as the coordination of Ci uptake, localized CO_2_ generation, and CO_2_ retention or recapture, each of which is adjusted according to external CO_2_ availability. First, Ci is accumulated through energy-dependent uptake steps, including ABC-type HCO_3_^−^ uptake at the plasma membrane and subsequent transfer across the chloroplast envelope and thylakoid membrane. Second, localized CA activity converts accumulated HCO_3_^−^ to CO_2_ in the immediate vicinity of Rubisco. Third, diffusion barriers and recapture systems retain or recover that CO_2_ long enough for fixation.

Under VLC conditions, *Chlamydomonas* induces the plasma-membrane ABC-type transporter HLA3, which contributes to HCO_3_^−^ uptake (Duanmu et al., 2009; Gao et al., 2015). HCO_3_^−^ then crosses the chloroplast envelope through LCIA, a chloroplast-envelope bicarbonate channel/transporter (Förster et al., 2023; Guo et al., 2026; Yamano et al., 2015), and is thought to enter the thylakoid lumen through bestrophin-like proteins BST1–3 (Mukherjee et al., 2019). In the pyrenoid-traversing thylakoid tubules, the α-type CA CAH3 dehydrates HCO_3_^−^ to CO_2_ near the Rubisco-rich matrix (Karlsson et al., 1998; Mackinder et al., 2017). A complementary recapture system is provided by the stromal β-CA-like LCIB–LCIC complex. LCIB relocalizes to the pyrenoid periphery under VLC conditions, where it is proposed to convert CO_2_ leaking from the fixation site back to HCO_3_^−^ and help maintain the stromal Ci pool (Kasili et al., 2023; Yamano et al., 2010, 2022). Correct pyrenoid-peripheral localization of LCIB depends on the starch sheath and LCIC (Toyokawa et al., 2020; Yamano et al., 2022).

Under LC conditions, when CO_2_ availability rises above the VLC range but remains limiting, Ci acquisition can pivot toward a CO_2_-uptake route involving the plasma-membrane protein LCI1 and the periplasmic α-type CA CAH1. LCI1 functions in active CO_2_ uptake and is proposed to act as a plasma-membrane CO_2_ channel (Kono et al., 2020; Kono and Spalding, 2020), whereas CAH1 converts extracellular HCO_3_^−^ to CO_2_ and thereby supports high Ci affinity, particularly under alkaline conditions (Shimamura et al., 2024).

The efficiency of these uptake routes depends on pyrenoid architecture. CAH3- and BST-enriched thylakoid tubules traverse the pyrenoid matrix, positioning lumenal HCO_3_^−^ dehydration close to Rubisco (Mackinder et al., 2017; Mukherjee et al., 2019; Hennacy et al., 2024). The starch sheath contributes to CCM performance by supporting LCIB localization and is predicted to reduce CO_2_ leakage from the Rubisco-rich matrix (Fei et al., 2022; Toyokawa et al., 2020). The SAGA1–MITH1 module generates matrix-traversing membranes, physically coupling lumenal Ci processing to the pyrenoid matrix (Hennacy et al., 2024).

Taken together, the *Chlamydomonas* CCM is best viewed as a spatially organized carbon-flux system. Its performance depends not on any single transporter, CA, or structural element, but on the coordination of Ci uptake, localized CO_2_ generation, leakage recapture, diffusion limitation, and pyrenoid architecture.

## Energy supply and metabolic integration in the CCM

In *Chlamydomonas*, operation of the CCM imposes an additional energetic demand under CO_2_-limiting conditions because Ci uptake, thylakoid lumen acidification, and localized CO_2_ generation all depend on photosynthetic energy supply. Meeting this demand while maintaining Calvin–Benson cycle activity requires alternative electron-transfer pathways that adjust the ATP/NADPH balance and sustain the proton motive force (Burlacot et al., 2022; Giordano et al., 2005; Raven et al., 2014).

Within the chloroplast, PGRL1-dependent cyclic electron flow around photosystem I and flavodiiron-protein-dependent pseudo-cyclic electron flow increase the proton motive force without producing additional NADPH. Burlacot et al. (2022) showed that these pathways are required to generate a low thylakoid lumen pH that is essential for CCM function. The resulting ΔpH supports ATP synthesis and creates a lumenal environment favorable for HCO_3_^−^ dehydration by CAH3. This interpretation is consistent with the thylakoid localization and CCM requirement of BST1–3 and CAH3, and with biochemical evidence that CAH3 is most active at the slightly acidic pH values expected in the illuminated thylakoid lumen (Benlloch et al., 2015; Karlsson et al., 1998; Mukherjee et al., 2019).

Energy supply must also be considered together with pyrenoid architecture. Modeling of the pyrenoid-based CCM indicates that efficient CO_2_ concentration requires localized CO_2_ generation, correct enzyme placement, and a physical barrier that limits CO_2_ leakage from the pyrenoid matrix (Fei et al., 2022). Thus, the energetic role of lumen acidification is inseparable from the spatial organization of CAH3, thylakoid tubules, and Rubisco within the pyrenoid. Recent reviews have emphasized the same principle as a key constraint for transferring pyrenoid-based CCMs into plants (Catherall et al., 2025).

Chloroplast-to-mitochondrion electron flow provides another route for balancing reductant and ATP demand. Burlacot et al. (2022) proposed that this pathway contributes to energizing non-thylakoid Ci uptake steps, probably by supporting respiratory ATP production. Consistent with this view, mitochondria relocate from a more central distribution toward the cell periphery during CCM induction, supporting a functional connection between respiration and CCM performance (Findinier et al., 2024). More recent quantitative work further showed that cyclic, pseudo-cyclic, and chloroplast-to-mitochondrion electron flows can compensate for one another, and that at least one of these alternative pathways is required to sustain efficient CO_2_ fixation (Peltier et al., 2024).

This mitochondrial response is itself regulated as part of the CCM acclimation program. Findinier et al. (2024) showed that mitochondrial movement toward the cell periphery occurs within approximately 90 min after transfer to VLC conditions, requires RNA and protein synthesis, and is regulated by CCM1/CIA5. Blocking mitochondrial electron transport in VLC-acclimated cells reduces Ci affinity, supporting a functional link between mitochondrial activity and CCM performance.

The energetic demand of the CCM is distributed across several mechanistically distinct steps. The clearest candidate for direct ATP-dependent Ci uptake is the plasma-membrane ABC-type HCO_3_^−^ transporter HLA3. By contrast, LCIA and BST1–3 are best described as bicarbonate channels or channel-like transport proteins, and their operation is expected to depend on electrochemical gradients rather than direct ATP hydrolysis. LCI1 is proposed to facilitate CO_2_ uptake at the plasma membrane, whereas CAH1 and CAH3 catalyze CO_2_/HCO_3_^−^ interconversion rather than consuming ATP directly. Thus, CEF and PCEF support the CCM primarily by generating ATP and thylakoid ΔpH for lumenal CAH3 activity, whereas CMEF and mitochondrial ATP production are proposed to support non-thylakoid uptake steps, including HLA3-dependent HCO_3_^−^ uptake.

Integration with central carbon metabolism is more nuanced than a simple photorespiration-suppression model. During CO_2_-limiting acclimation, CCM and photorespiratory genes are simultaneously induced, and both programs are linked to CCM1/CIA5-dependent regulation. Physiological analyses further show that photorespiration remains active when the CCM is operational, indicating that CCM induction reduces but does not eliminate oxygenation pressure around Rubisco (Dao et al., 2025). Inefficient CO_2_ retention would also increase the demand for Ci recapture and re-entry into the concentrating cycle, thereby increasing the energetic burden of the CCM as a whole.

Taken together, energy supply in the *Chlamydomonas* CCM is distributed across multiple pathways rather than assigned to a single ATP source. It is a flexible network that couples thylakoid ΔpH, alternative electron flow, chloroplast–mitochondrial cooperation, pyrenoid architecture, and central carbon metabolism. This energetic network provides the foundation on which the regulatory circuits discussed below can tune CCM activity according to CO_2_ availability and cellular metabolic state.

## Multilayered regulation of the CCM

In *Chlamydomonas*, CCM activity is coordinated by regulatory layers that act over different timescales, from rapid changes in protein localization and post-translational state to slower transcriptional induction and repression.

A central transcriptional layer is controlled by CCM1/CIA5, a Zn-finger-containing regulator required for the induction of many CCM genes under CO_2_-limiting conditions. These include genes encoding Ci transport proteins and CAs such as *HLA3*, *LCIA*, *LCIB*, *LCIC*, *CAH1*, and *CAH3* (Fang et al., 2012; Fukuzawa et al., 2001; Miura et al., 2004; Xiang et al., 2001; Yamano et al., 2008). Earlier studies did not detect direct DNA binding by CCM1/CIA5, but recent *in vitro* work using purified full-length CIA5 demonstrated specific binding to a GC-rich motif found in promoters of CIA5-dependent genes (Chen and Spalding, 2026). This provides the first *in vitro* biochemical evidence that CCM1/CIA5 can directly recognize promoter DNA. Recent work further indicates that CCM1/CIA5 coordinates part of the photorespiratory response to CO_2_ limitation, suggesting that it governs a broader carbon-acclimation state rather than only a canonical CCM regulon (Dao et al., 2025). The primary CO_2_/HCO_3_^−^ sensor upstream of CCM1/CIA5 remains unknown.

Downstream of CCM1/CIA5, branch-specific transcriptional regulators further partition the response to CO_2_ limitation. The Myb-type transcription factor LCR1 regulates *CAH1* and other target genes in response to CO_2_ limitation, indicating that CCM1/CIA5-dependent induction is subdivided into more specific transcriptional modules (Yoshioka et al., 2004). The physiological importance of CAH1 in Ci acquisition has recently been reinforced by genetic analysis (Shimamura et al., 2024).

A second layer involves spatial reorganization of CCM components. LCIB–LCIC localization provides a useful marker for distinguishing LC and VLC states. LCIB remains largely dispersed in the chloroplast stroma under HC and LC conditions but relocalizes to the pyrenoid periphery under VLC conditions. This relocalization is proposed to promote recapture of CO_2_ leaking from the fixation site and depends on the starch sheath (Toyokawa et al., 2020; Yamano et al., 2010, 2022). This CO_2_-dependent change in LCIB–LCIC localization is included in the HC/LC/VLC switch shown in **Figure 1B**. In parallel, BST1–3 and CAH3 are positioned within pyrenoid-traversing thylakoid tubules, placing HCO_3_^−^ transport and lumenal CO_2_ generation near the Rubisco-rich matrix (Mackinder et al., 2017; Mukherjee et al., 2019). At the level of pyrenoid architecture, SAGA1 and MITH1 generate the matrix-traversing membranes themselves. Mutants lacking either factor lose these membranes and show growth defects under CO_2_-limiting conditions (Hennacy et al., 2024).

A third regulatory layer is provided by CAS-dependent Ca^2+^-linked signaling. CAS is a chloroplast Ca^2+^-binding protein that changes localization in response to CO_2_ conditions and regulates nuclear CCM gene expression through a chloroplast-to-nucleus signal (Wang et al., 2016). SAGA1 also links pyrenoid architecture to CAS-dependent signaling. Disruption of SAGA1 impairs CAS localization to the pyrenoid and weakens CAS-dependent expression of nuclear genes encoding Ci transporters, indicating that pyrenoid structure itself participates in CCM regulation (Shimamura et al., 2023). The simplified CAS localization pattern shown in **Figure 1B** highlights this regulatory connection between pyrenoid-associated chloroplast signaling and nuclear CCM gene expression.

Genetic interaction and mutant-combination studies further support this modular view. Analyses of HLA3 and LCIA define cooperative plasma-membrane and chloroplast-envelope steps for HCO_3_^−^ uptake, whereas studies of LCIB and LCIA revealed condition-dependent contributions to LC and VLC acclimation (Wang and Spalding, 2014; Yamano et al., 2015). Starch-sheath-defective and LCIC-related mutants demonstrate that pyrenoid architecture is required for correct LCIB localization (Toyokawa et al., 2020; Yamano et al., 2022). SAGA1 further links pyrenoid architecture to CAS-dependent nuclear gene expression, because SAGA1 disruption impairs CAS localization and weakens CAS-dependent expression of nuclear genes encoding Ci transporters (Shimamura et al., 2023). These studies show that transport, pyrenoid structure, and nuclear regulation are genetically and functionally coupled.

Post-translational regulation provides another layer of control. Early phosphoproteomic work showed that CO_2_ limitation alters phosphorylation of multiple CCM-associated proteins (Turkina et al., 2006). A functional precedent is CAH3 phosphorylation. Acclimation to CO_2_ limitation promotes CAH3 phosphorylation, partial activation, and redistribution within thylakoid membranes, indicating that phosphorylation can tune both enzymatic activity and spatial organization of CCM components (Blanco-Rivero et al., 2012). More recently, the pyrenoid-localized kinase KEY1 was shown to phosphorylate EPYC1 at Rubisco-binding sites. This weakens Rubisco–EPYC1 interactions and controls pyrenoid size, number, and dissolution (He et al., 2026). Notably, KEY1 is induced by CO_2_-limiting stress in a CCM1/CIA5-dependent manner, indicating that transcriptional induction can feed directly into condensate-level control of pyrenoid architecture (Shimamura et al., 2026). A key unresolved question is whether CO_2_-limiting acclimation also induces phosphatase activities that reset EPYC1 phosphorylation and restore pyrenoid condensability.

In addition to these inductive regulatory layers, the CCM is actively repressed under HC conditions. The nuclear protein CBP1 interacts with CCM1/CIA5 and represses the expression of 27 of 41 CCM1-dependent genes under HC conditions. In *cbp1* mutants, CAH1 and LCIB protein levels increase and Ci affinity rises even under HC conditions. These findings indicate that CBP1 prevents unnecessary CCM activation when CO_2_ is abundant, thereby contributing to energy conservation (Shimamura et al., 2026). The CobW/CobW_C GTP-binding metallochaperone module in CBP1 raises the possibility that metal-dependent regulation contributes to CCM repression. This HC repression state, together with the release of CCM1/CIA5-dependent induction under CO_2_-limiting conditions, is schematized in **Figure 1B**.

How far this regulatory architecture extends to other green algae remains an important open question. The *Chlamydomonas* transcriptional regulator CCM1/CIA5 appears to be a lineage-restricted component rather than a broadly conserved green-plant regulator. A CCM1/CIA5 ortholog has been reported in *Volvox carteri*, where cells grown under CO_2_-limiting conditions show increased Ci affinity and the *Volvox* protein shares 51% amino-acid identity with *Chlamydomonas* CCM1/CIA5, including conservation of key N-terminal and zinc-finger-related regions (Yamano et al., 2011; Brueggeman et al., 2012). Genomic analyses have also annotated CIA5/CCM1 among *Chlamydomonas* CCM-associated orthologs in the trebouxiophyte *Coccomyxa subellipsoidea* C-169, although functional conservation of this regulator has not been demonstrated (Blanc et al., 2012). Thus, CCM1/CIA5-dependent transcriptional control may represent a specialized regulatory solution in a subset of chlorophytes.

The conservation of LCR1-type branch-specific regulation is also uncertain. LCR1 is a divergent 1R-Myb transcription factor in *Chlamydomonas*, and orthology outside closely related green algae should be inferred cautiously without dedicated Myb-family phylogenetic analysis (Yoshioka et al., 2004; Brueggeman et al., 2012).

By contrast, CAS is a more broadly conserved chloroplast Ca^2+^ signaling component. CAS functions in *Chlamydomonas* CCM regulation and photoacclimation, whereas *Arabidopsis* CAS is required for Ca^2+^-dependent stomatal regulation and chloroplast-mediated signaling (Nomura et al., 2008; Petroutsos et al., 2011; Wang et al., 2016; Weinl et al., 2008). The *Chlamydomonas* CCM therefore appears to combine lineage-restricted transcriptional regulation with chloroplast Ca^2+^ signaling modules that have functional counterparts in both *Chlamydomonas* and land plants.

## Conclusions and future perspectives

Taken together, recent work supports a view of the *Chlamydomonas* CCM as a coupled system in which Ci transport, energy supply, pyrenoid architecture, and regulation are inseparable. Flexible Ci uptake routes, lumen acidification, chloroplast–mitochondrial cooperation, CO_2_ retention, and multilayered control circuits together determine whether Rubisco remains supplied with high local CO_2_ under fluctuating environments. At the same time, conservation of individual regulatory modules is uneven across green algae. CCM1/CIA5-dependent transcriptional regulation, and possibly LCR1-type branch-specific control, appear relatively restricted, whereas CAS-linked chloroplast Ca^2+^ signaling has functional counterparts in both *Chlamydomonas* and land plants.

The next challenge is to identify how the system switches between carbon-replete HC conditions and CO_2_-limiting LC/VLC states. The upstream CO_2_/HCO_3_^−^ sensor for CCM1/CIA5 remains unknown, and one plausible possibility is that sensing is mediated by a multi-component signaling module rather than by a single receptor. As a conceptual analogy, Arabidopsis guard cells show that CO_2_/HCO_3_^−^ sensing can be distributed across interacting proteins. The HT1–MPK4/12 module functions as a primary CO_2_/HCO_3_^−^ sensor, and CA4 can regulate the SLAC1 anion channel through a binding function that is separable from CA catalytic activity (Takahashi et al., 2022; Xia et al., 2026). These findings raise the possibility that *Chlamydomonas* CO_2_ sensing may also involve modular interactions among CAs, kinases, and membrane transport proteins, although no equivalent upstream sensor has yet been identified in *Chlamydomonas*.

KEY1 now provides a mechanistic entry point into condensate-level CCM regulation. Because KEY1 is induced by CO_2_-limiting stress in a CCM1/CIA5-dependent manner (Shimamura et al., 2026), transcriptional induction provides a route into pyrenoid phase control. Mechanistically, KEY1 phosphorylates EPYC1 at Rubisco-binding sites, weakens Rubisco–EPYC1 interactions, and regulates pyrenoid size, number, and dissolution (He et al., 2026). This raises the reciprocal question of whether CO_2_-limiting acclimation also induces phosphatase activities that reset EPYC1 phosphorylation and restore pyrenoid condensability. No phosphatase has yet been shown to dephosphorylate EPYC1 or to promote pyrenoid condensation. Nevertheless, identifying phosphatase activities induced under CO_2_-limiting conditions is now a testable way to examine whether pyrenoid assembly is regulated by reversible phosphorylation. Time-resolved phosphoproteomics, acute kinase and phosphatase perturbations, and *in vitro* reconstitution of Rubisco–EPYC1–KEY1 reactions with candidate phosphatases could reveal whether pyrenoid assembly is actively tuned by reversible phosphorylation cycles.

The distinction between transferring CCM components and recreating their regulatory logic is important for engineering. Current efforts to build pyrenoid-based CCMs in plants have focused mainly on transferring or reconstituting structural and catalytic components, such as Rubisco condensation, pyrenoid matrix formation, CO_2_-retention structures, transport steps, and CAs (Atkinson et al., 2020; Fei et al., 2022; Catherall et al., 2025). These approaches establish the feasibility of component transfer, but they do not yet recreate the regulatory logic by which the *Chlamydomonas* CCM is switched on, restrained, spatially reorganized, and energetically coordinated according to CO_2_ availability. Thus, a “switchable CCM” should be viewed at present as a design principle emerging from *Chlamydomonas* regulation rather than as an already demonstrated crop-engineering module. Future designs may need to combine pyrenoid components with plant-compatible regulatory systems, such as inducible promoters, carbon- or energy-responsive sensors, and reversible phosphorylation switches, so that CCM activity can track carbon status, light-dependent energy supply, and developmental context. For heterologous photosynthetic systems, the ability to switch CCM activity in response to carbon and energy status may be as critical as the choice of individual transporters, CAs, or pyrenoid structural components.
